# Cytokeratin 5/6 and cytokeratin 8/18 expression in triple negative breast cancers: clinicopathologic significance in South-Asian population

**DOI:** 10.1186/s13104-018-3477-4

**Published:** 2018-06-08

**Authors:** Atif Ali Hashmi, Samreen Naz, Shumaila Kanwal Hashmi, Zubaida Fida Hussain, Muhammad Irfan, Syed Muhammad Abu Bakar, Naveen Faridi, Amir Khan, Muhammad Muzzammil Edhi

**Affiliations:** 1 Liaquat National Hospital and Medical College, Karachi, Pakistan; 2 CMH Institute of Medical Sciences, Multan, Pakistan; 3 Kandahar University, Kandahar, Afghanistan; 4 Brown University, Providence, RI USA

**Keywords:** Triple negative breast cancer, CK5/6, CK8/18, Basal cytokeratins, Luminal cytokeratins

## Abstract

**Objective:**

Cytokeratin 5/6 and Cytokeratin 8/18 are basal and luminal markers of breast cancer and they have pathological and prognostic significance in breast cancer. We performed Cytokeratin 5/6 and CK8/18 immunohistochemistry on 150 cases of triple negative breast cancers and association with various clinicopathological features was evaluated.

**Results:**

Positive CK5/6 expression was noted in 8% (12 cases) of TNBC while 2.4% (4 cases) showed focal positive (< 10%) and 89.3% (134) were negative with CK5/6. Complete loss of CK8/18 expression was seen in 4.7% (7 cases) while 32.7% (49 cases) revealed focal loss of CK8/18 and 62.7% (94 cases) showed intact normal expression of CK8/18. No significant association of CK5/6 and CK8/18 with various clinicopathological parameters was observed. We found a low expression of basal cytokeratin (CK5/6) in TNBC our studied population, while loss/altered expression of CK8/18 in approximately 38% of TNBC. Although no prognostic relevance of these finding was noted in our study, however these findings are different from those reported in literature in other parts of the world. Therefore we suggest a more through immunohistochemical and genomic profiling of TNBC in our population for better understanding of this disease in this part of the world.

**Electronic supplementary material:**

The online version of this article (10.1186/s13104-018-3477-4) contains supplementary material, which is available to authorized users.

## Introduction

Triple negative breast cancer (TNBC) is a heterogeneous group of breast cancer accounting for 15–20% of newly diagnosed breast cancer [1–3]. TNBC is a clinically defined term with lack of immunohistochemistry (IHC) expression of Estrogen receptor (ER), progesterone receptor (PR) and human epidermal growth factor receptor 2 (her2neu) according to College of American Pathologist (CAP)/ASCO guidelines [4, 5]. This clinically defined subtype of breast cancer also comprise basal like molecular subtype, however triple negative and basal breast cancers are not synonymous and there is substantial overlap and heterogeneity among these two groups. For example in a study; 71% of TNBC were found to be of basal subtype and conversely 77% basal tumors were triple negative by IHC analysis [6]. However, in resource limited countries like Pakistan where molecular testing is not widely available, relying on IHC expression of cytokeratins can be considered acceptable. Basal like breast cancers usually express basal cytokeratins like CK5/6, CK14 and CK17 [7]. CK5/6 is the most useful and most important marker for the identification of basal subgroup of TNBC [8]. CK8/18 is another marker which can be called luminal marker as it represents normal luminal epithelial differentiation [8] and thus loss of CK8/18 expression in TNBC may have pathological relevance. Expression of these markers in TNBC has not been widely studied in our population, therefore in the present study we aimed to evaluate the expression of CK 5/6 and CK8/18 in TNBC in our population and their prognostic significance.

## Main text

### Methods

Total 150 cases of TNBC were selected from records of pathology department archives. The surgical specimens included modified radical mastectomies, simple mastectomies and wide local excisions with sentinel lymph node dissection. All patients underwent surgeries at Liaquat National hospital, Karachi from January 2008 till December 2013 over a period of 6 years. Study was approved by research and ethical review committee of the institution and informed consent was taken from all patients at the time of surgery. Hematoxylin and eosin stained slides and paraffin blocks of cases were retrieved and new sections were cut where necessary. Slides of all cases were reviewed by two senior histopathologists and pathologic characteristics like histologic type, tumor grade, T-stage, N-stage, lymphovascular invasion, necrosis, fibrosis, lymphocytic infiltration (tumor infiltrating lymphocytes), presence of insitu component, pagetoid spread and dermal lymphatic invasion were evaluated. Clinical records of all patients were also reviewed from institutional records to evaluate patient’s age, history of radiation and chemotherapy and recurrence status. Moreover, representative blocks of each case were selected and ER, PR and Her2neu immunohistochemistry were performed to reconfirm triple negative status.

ER, PR, Her2neu and Ki67 IHC were performed using DAKO antibodies as under, with EnVision™ FLEX, high pH DAKO kit according to manufacturer’s protocol.

1. FLEX monoclonal rabbit anti-human estrogen receptor alpha, clone EP1.
2. FLEX monoclonal mouse anti-human progesterone receptor clone PgR 636.
3. Polyclonal rabbit anti-human c-erbB-2 oncoprotein.
4. FLEX monoclonal mouse anti-human Ki67 antigen clone MIB-1.

Nuclear staining of ER and PR in > 1% of cancer cells was considered positive. For her2neu, cases were scored 1+, 2+ and 3+ according to CAP guidelines. For equivocal cases (2+), FISH was performed using Path Vysion Her2neu probe kit according to manufacturer’s protocols. Results were expressed as ratio of her2neu signals as compared to CEP17 signals according to ASCO/CAP guidelines.

Ki67 immunostaining was recorded as continuous variables based on proportion of positive stained cancer cells. Furthermore on the basis of Ki67 index, cases were categorized into < 15, 16–24, 25–44 and > 44% ki67 index categories.

CK5/6 IHC was performed by using FLEX Monoclonal Mouse Anti-human Cytokeratin 5/6, clone D5/16 B4 by DAKO envision method according to manufacturers protocol. Intermediate to strong cytoplasmic and membranous staining in more than 10% cells was considered positive. Weak to intermediate staining in < 10% was taken as focal positive, while no staining was considered as negative (Additional file 1: Figure S1).

CK 8/8 IHC was performed using monoclonal mouse anti-human Cytokeratin 5/6, clone D5/16 B4, according to DAKO envision method. Intermediate to strong cytoplasmic and membranous staining in more than 50% cells was considered positive. Weak to intermediate staining (cytoplasmic, with loss of membrane reactivity) in 10–50% was taken as focal positive, while no staining or weak staining in < 10% was considered as negative (Additional file 1: Figure S1).

Recurrence status and follow-up were evaluated by reviewing hospital medical record. Overall survival was taken as time from surgical excision till death or last follow-up and disease free survival was defined as time between surgical excision and local recurrence or distant metastasis, death or last follow-up.

Statistical package for social sciences (SPSS 21) was used for data compilation and analysis. Mean and standard deviation were calculated for quantitative variables. Frequency and percentage were calculated for qualitative variables. Chi square was applied to determine association. Student t test or Mann whitney test were applied to compare difference in means among groups. *P* value ≤ 0.05 as significant. Survival curves were plotted using Kaplan–Meier method and the significance of difference between survival curves were determined using log-rank ratio. P-value ≤ 0.05 was taken as significant.

### Results

Mean age of the patients involved in the study was 48.9 years and most common age group was 31–50 years. Most of the patients presented at stage T2 with a high mean ki67 index i.e. 46.9%. 42.7% of cases had nodal metastasis. Although 84% cases were of conventional invasive ductal carcinoma, NST; however a significant proportion of cases were of metaplastic histology (9.3%). Majority cases were of high grade (86.7% grade III). Most tumors show lymphocytic infiltration and necrosis. Most of the tumors lack insitu component (61%). Local recurrence or late distant metastasis was noted in 17.8% of cases (Table 1).

**Table 1:** Descriptive statistics of studied population

	n (%)
Age (years)^a^	48.85 ± 11.49
Age groups (years)	
≤ 30	5 (3.3)
31–50	84 (56)
> 50	61 (40.7)
Tumor size (unit)^a^	4.01 ± 1.99
Tumor stage	
T1	26 (17.3)
T2	79 (52.7)
T3/T4	45 (30)
Ki67 index (%)	46.89 ± 23.88
ki67 index groups (%)	
≤ 15	17 (11.3)
16–24	8 (5.3)
25–44	45 (30)
> 44	80 (53.3)
Nodal status	
Positive	64 (42.7)
Negative	86 (57.3)
Nodal stage	
No	88 (58.7)
N1	30 (20)
N2	13 (8.7)
N3	19 (12.7)
Histological subtypes	
IDC	127 (84.7)
Papillary	6 (4)
Medullary	1 (0.7)
Metaplastic	14 (9.3)
Mixed	2 (1.3)
Tumor grade	
Grade-I	1 (0.7)
Grade-II	19 (12.7)
Grade-III	130 (86.7)
Lymphocytic infiltration	
Absent	15 (10)
Moderate	110 (73.3)
Severe	25 (16.7)
Lymphovascular invasion	
Present	36 (24)
Absent	114 (76)
Dermal lymphatic invasion	
Present	10 (6.7)
Absent	140 (93.3)
Type of surgery	
Modified radical mastectomy	94 (62.7)
Simple mastectomy with sentinel lymph node dissection	42 (28)
Wide local excision	14 (9.3)
Necrosis	
Absent	21 (14)
Moderate	90 (60)
Severe	39 (26)
Fibrosis	
Mild	42 (28)
Moderate	88 (58.7)
Severe	20 (13.3)
Insitu component	
Present	58 (38.7)
Absent	92 (61.3)
Pagetoid spread	
Present	2 (1.3)
Absent	148 (98.7)
Perinodal extension	
Present	30 (20)
Absent	120 (80)
Adjuvant chemotherapy (n = 101)	
Yes	98 (97)
No	3 (3)
Adjuvant radiotherapy (n = 101)	
Yes	69 (68.3)
No	32 (31.7)
Reoccurrence (n = 101)	
Yes	18 (17.8)
No	83 (82.2)

^a^Mean ± SD

Positive (> 10%) CK5/6 expression was noted in 8% (12 cases) of TNBC while 2.4% (4 cases) showed focal positive (< 10%) and 89.3% (134) were negative with CK5/6. Complete loss of CK8/18 expression was seen in 4.7% (7 cases) while 32.7% (49 cases) revealed focal loss of CK8/18 and 62.7% (94 cases) showed intact normal expression of CK8/18. No significant association of CK5/6 and CK8/18 with various clinicopathological parameters was observed (Tables 2 and 3). Similarly no significant association of CK 5/6 and CK8/18 with recurrence status of the patients was noted (Additional file 2: Figure S2).

**Table 2:** Association of CK 5/6 expression with clinicopathologic features of triple negative breast cancer

	n (%)				P-value
	No loss of expression (n = 12)	Complete loss of expression (n = 4)	Focal loss of expression (n = 134)	Total (n = 150)	
Age groups (years)					
≤ 30	0 (0)	0 (0)	5 (3.7)	5 (3.3)	0.907
31–50	8 (66.7)	2 (50)	74 (55.2)	84 (56)	
> 50	4 (33.3)	2 (50)	55 (41)	61 (40.7)	
Tumor stage					
T1	1 (8.3)	0 (0)	25 (18.7)	26 (17.3)	0.690
T2	6 (50)	2 (50)	71 (53)	79 (52.7)	
T3/T4	5 (41.7)	2 (50)	38 (28.4)	45 (30)	
ki67 index groups (%)					
≤ 15	3 (25)	1 (25)	13 (9.7)	17 (11.3)	0.272
16–24	0 (0)	0 (0)	8 (6)	8 (5.3)	
25–44	1 (8.3)	1 (25)	43 (32.1)	45 (30)	
> 44	8 (66.7)	2 (50)	70 (52.2)	80 (53.3)	
Nodal status					
Positive	4 (33.3)	2 (50)	58 (43.3)	64 (42.7)	0.763
Negative	8 (66.7)	2 (50)	76 (56.7)	86 (57.3)	
Nodal stage					
No	8 (66.7)	2 (50)	78 (58.2)	88 (58.7)	0.232
N1	1 (8.3)	1 (25)	28 (20.9)	30 (20)	
N2	3 (25)	0 (0)	10 (7.5)	13 (8.7)	
N3	0 (0)	1 (25)	18 (13.4)	19 (12.7)	
Histological subtypes					
IDC	9 (75)	4 (100)	114 (85.1)	127 (84.7)	0.419
Papillary	0 (0)	0 (0)	6 (4.5)	6 (4)	
Medullary	0 (0)	0 (0)	1 (0.7)	1 (0.7)	
Metaplastic	2 (16.7)	0 (0)	12 (9)	14 (9.3)	
Mixed	1 (8.3)	0 (0)	1 (0.7)	2 (1.3)	
Tumor grade					
Grade-I	0 (0)	0 (0)	1 (0.7)	1 (0.7)	1.000
Grade-II	1 (8.3)	0 (0)	18 (13.4)	19 (12.7)	
Grade-III	11 (91.7)	4 (100)	115 (85.8)	130 (86.7)	
Lymphocytic infiltration					
Absent	0 (0)	0 (0)	15 (11.2)	15 (10)	0.689
Moderate	9 (75)	4 (100)	97 (72.4)	110 (73.3)	
Severe	3 (25)	0(0)	22 (16.4)	25 (16.7)	
Lymphovascular invasion					
Present	1 (8.3)	1 (25)	34 (25.4)	36 (24)	0.466
Absent	11 (91.7)	3 (75)	100 (74.6)	114 (76)	
Dermal lymphatic invasion					
Present	1 (8.3)	1 (25)	8 (6)	10 (6.7)	0.172
Absent	11 (91.7)	3 (75)	126 (94)	140 (93.3)	
Type of surgery					
Modified radical mastectomy	10 (83.3)	2 (50)	82 (61.2)	94 (62.7)	0.522
Simple mastectomy with sentinel lymph node dissection	2 (16.7)	2 (50)	38 (28.4)	42 (28)	
Wide local excision	0 (0)	0 (0)	14 (10.4)	14 (9.3)	
Necrosis					
Absent	2 (16.7)	1 (25)	18 (13.4)	21 (14)	0.507
Moderate	7 (58.3)	1 (25)	82 (61.2)	90 (60)	
Severe	3 (25)	2 (50)	34 (25.4)	39 (26)	
Fibrosis					
Mild	4 (33.3)	0 (0)	38 (28.4)	42 (28)	0.167
Moderate	8 (66.7)	2 (50)	78 (58.2)	88 (58.7)	
Severe	0 (0)	2 (50)	18 (13.4)	20 (13.3)	
Insitu component					
Present	2 (16.7)	3 (75)	53 (39.6)	58 (38.7)	0.098
Absent	10 (83.3)	1 (25)	81 (60.4)	92 (61.3)	
Pagetoid spread					
Present	0 (0)	0 (0)	2 (1.5)	2 (1.3)	1.000
Absent	12 (100)	4 (100)	132 (98.5)	148 (98.7)	
Perinodal extension					
Present	2 (16.7)	1 (25)	27 (20.1)	30 (20)	1.000
Absent	10 (83.3)	3 (75)	107 (79.9)	120 (80)	

Chi Square test applied

P-value ≤ 0.05, considered as significant

**Table 3:** Association of CK 5/6 expression with clinicopathologic features of triple negative breast cancer

	n (%)				P-value
	No loss of expression (n = 94)	Complete loss of expression (n = 7)	Focal loss of expression (n = 49)	Total (n = 150)	
Age groups (years)					
≤ 30	1 (1.1)	1 (14.3)	3 (6.1)	5 (3.3)	0.061
31–50	49 (52.1)	4 (57.1)	31 (63.3)	84 (56)	
> 50	44 (46.8)	2 (28.6)	15 (30.6)	61 (40.7)	
Tumor stage					
T1	20 (21.3)	2 (28.6)	4 (8.2)	26 (17.3)	0.227
T2	47 (50)	4 (57.1)	28 (57.1)	79 (52.7)	
T3/T4	27 (28.7)	1 (14.3)	17 (34.7)	45 (30)	
ki67 index groups (%)					
≤ 15	8 (8.5)	2 (28.6)	7 (14.3)	17 (11.3)	0.495
16–24	6 (6.4)	0 (0)	2 (4.1)	8 (5.3)	
25–44	32 (34)	1 (14.3)	12 (24.5)	45 (30)	
> 44	48 (51.1)	4 (57.1)	28 (57.1)	80 (53.3)	
Nodal status					
Positive	40 (42.6)	4 (57.1)	20 (40.8)	64 (42.7)	0.715
Negative	54 (57.4)	3 (42.9)	29 (59.2)	86 (57.3)	
Nodal stage					
No	55 (58.5)	3 (42.9)	30 (61.2)	88 (58.7)	0.152
N1	16 (17)	3 (42.9)	11 (22.4)	30 (20)	
N2	7 (7.4)	0 (0)	6 (12.2)	13 (8.7)	
N3	16 (17)	1 (14.3)	2 (4.1)	19 (12.7)	
Histological subtypes					
IDC	83 (88.3)	6 (85.7)	38 (77.6)	127 (84.7)	0.138
Papillary	5 (5.3)	0 (0)	1 (2)	6(4)	
Medullary	1 (1.1)	0 (0)	0 (0)	1 (0.7)	
Metaplastic	4 (4.3)	1 (14.3)	9 (18.4)	14 (9.3)	
Mixed	1 (1.1)	0 (0)	1 (2)	2 (1.3)	
Tumor grade					
Grade-I	1 (1.1)	0 (0)	0 (0)	1 (0.7)	0.437
Grade-II	15 (16)	0 (0)	4 (8.2)	19 (12.7)	
Grade-III	78 (83)	7 (100)	45 (91.8)	130 (86.7)	
Lymphocytic infiltration					
Absent	9 (9.6)	0 (0)	6 (12.2)	15 (10)	0.585
Moderate	72 (76.6)	5 (71.4)	33 (67.3)	110 (73.3)	
Severe	13 (13.8)	2 (28.6)	10 (20.4)	25 (16.7)	
Lymphovascular invasion					
Present	26 (27.7)	2 (28.6)	8 (16.3)	36 (24)	0.261
Absent	68 (72.3)	5 (71.4)	41 (83.7)	114 (76)	
Dermal lymphatic invasion					
Present	7 (7.4)	0 (0)	3 (6.1)	10 (6.7)	1.000
Absent	87 (92.6)	7 (100)	46 (93.9)	140 (93.3)	
Type of surgery					
Modified radical mastectomy	59 (62.8)	4 (57.1)	31 (63.3)	94 (62.7)	0.806
Simple mastectomy with sentinel lymph node dissection	25 (26.6)	2 (28.6)	15 (30.6)	42 (28)	
Wide local excision	10 (10.6)	1 (14.3)	3 (6.1)	14 (9.3)	
Necrosis					
Absent	14 (14.9)	1 (14.3)	6 (12.2)	21 (14)	0.459
Moderate	58 (61.7)	5 (71.4)	27 (55.1)	90 (60)	
Severe	22 (23.4)	1 (14.3)	16 (32.7)	39 (26)	
Fibrosis					
Mild	22 (23.4)	2 (28.6)	18 (36.7)	42 (28)	0.101
Moderate	58 (61.7)	5 (71.4)	25 (51)	88 (58.7)	
Severe	14 (14.9)	0 (0)	6 (12.2)	20 (13.3)	
Insitu component					
Present	42 (44.7)	3 (42.9)	13 (26.5)	58 (38.7)	0.588
Absent	52 (55.3)	4 (57.1)	36 (73.5)	92 (61.3)	
Pagetoid spread					
Present	2 (2.1)	0 (0)	0 (0)	2 (1.3)	0.078
Absent	92 (97.9)	7 (100)	49 (100)	148 (98.7)	
Perinodal extension					
Present	24 (25.5)	1 (14.3)	5 (10.2)	30 (20)	0.614
Absent	70 (74.5)	6 (85.7)	44 (89.8)	120 (80)	

Chi Square test applied

P-value ≤ 0.05, considered as significant

### Discussion

In the present study we assessed expression of one basal (CK5/6) and one luminal marker (CK8/18) in TNBC in our population and found that; 8 and 2.7% of TNBC showed positive and focal positive expression of CK5/6 respectively and 4.7 and 32.7% of TNBC revealed complete loss and focal loss of CK 8/18 expression respectively. In all those cases in which focal loss CK8/18 was noted, there was loss of membrane reactivity while cytoplasmic staining was retained in few cells. However no significant association was observed between these abnormal expression patterns of cytokeratins with various clinicopathological and prognostic parameters of TNBC in our population. Breast cancer is one of the commonest malignancies in South Asian population especially in young age [9–11].

Gene expression profiling defines basal breast cancers as those exhibiting basal clusters of genes that include EGFR, basal cytokeratin 5/6, C–kit, proliferation cluster and low expression of Her2neu and hormone receptor related genes [12–14]. However basal breast cancer is not a single entity and it includes several subtypes including two basal like subtypes (BL1 and BL2), mesenchymal, mesenchymal stem-like, immunomodulatory and luminal androgen subtypes [15]. In addition to that interferon rich and claudin-low subtypes have also been defined [16, 17]. Subtyping of basal breast cancers is not recommended by ASCO/CAP. CK 5/6 expression varies from 24 to 72% in TNBC as reported in previous studies [18, 19]. Other researchers have also proposed prognostic significance of CK 5/6 in node negative breast cancers [20]. Nielson et al. suggested that CK5/6 positive breast cancer have worse prognosis independent of tumor grade, T-stage and hormonal/Her2neu status [21]. Similarly in another study it was proposed that CK5/6 is marker of shorter disease free survival, independent of other prognostic factors of breast cancer [8]. Inanc et al. reported 50.5% expression of CK5/6 with positive correlation of CK5/6 TNBC with nodal metastasis and tumor size [22]. However, in contrast to these studies we found a low expression of CK5/6 expression in TNBC in our studied population, which may represent a different genomic profile of TNBC in our population, which needs to be explored in further studies. Prognostic role of CK5/6 has been proposed in other cancers of the body [23].

CK8/18 is a luminal cytokeratin mainly expressed in breast epithelium in a membranous and cytoplasmic pattern. Cimpean et al. defined these different patterns of CK8/18 in breast cancer; diffuse cytoplasmic, membranous and combined cytoplasmic and membranous [19].Some researchers have proposed that loss of CK8/18 expression/low CK8/18 is associated with worse prognosis and high risk of metastasis [24, 25]. However we did not find any prognostic significance of loss of CK8/18 expression with various prognostic factors and recurrence in TNBC.

We found that 32.7% of TNBC show focal loss of expression CK8/18. In all these cases there was loss of membranous reactivity of CK8/18 while cytoplasmic staining was retained in few cells. Aiad et al. in a study involving 70 cases of breast cancer found abnormal expression of CK8/18 (cytoplasmic) in 70% of Egyptian breast cancer, which suggest a lot of heterogeneity in cytokeratin expression of breast cancer in different parts of world [26].

## Limitations

One of the major limitations of our study was only 2 immuno-markers were performed, therefore we suggest a more through IHC and genomic profiling of TNBC in our population for better understanding of this disease in this part of the world. One of the possible reasons of discrepancy found in the results of our study with that of the previously reported literature could be due the fact that tumors generally exhibit a lot of heterogeneity and we performed IHC stains on one representative block. On the other hand; despite standardization, sample under or over fixation and technical issues may also be responsible for these discrepancies.

## Additional files

**Additional file 1: Figure S1.** CK5/6 and CK 8/18 expression in triple negative breast cancer.

**Additional file 2: Figure S2.** Kalpien-Meier curve (disease free survival) for CK 8/18 expression in triple negative breast cancer.

## Abbreviations

TNBC: triple negative breast cancer
IHC: immunohistochemistry
ER: estrogen receptor
PR: progesterone receptor
her2neu: human epidermal growth factor receptor 2
CAP: College of American Pathologist
