# Supplementary material for: Cytokeratin 5/6 and cytokeratin 8/18 expression in triple negative breast cancers: clinicopathologic significance in South-Asian population
Source: BMC Res Notes. 2018 Jun 8;11:372. doi: 10.1186/s13104-018-3477-4 (PMC5994131; doi:10.1186/s13104-018-3477-4)
Supplement: Supplementary file 1 — Additional file 1: Figure S1. CK5/6 and CK 8/18 expression in triple negative breast cancer. [file 13104_2018_3477_MOESM1_ESM.pdf]

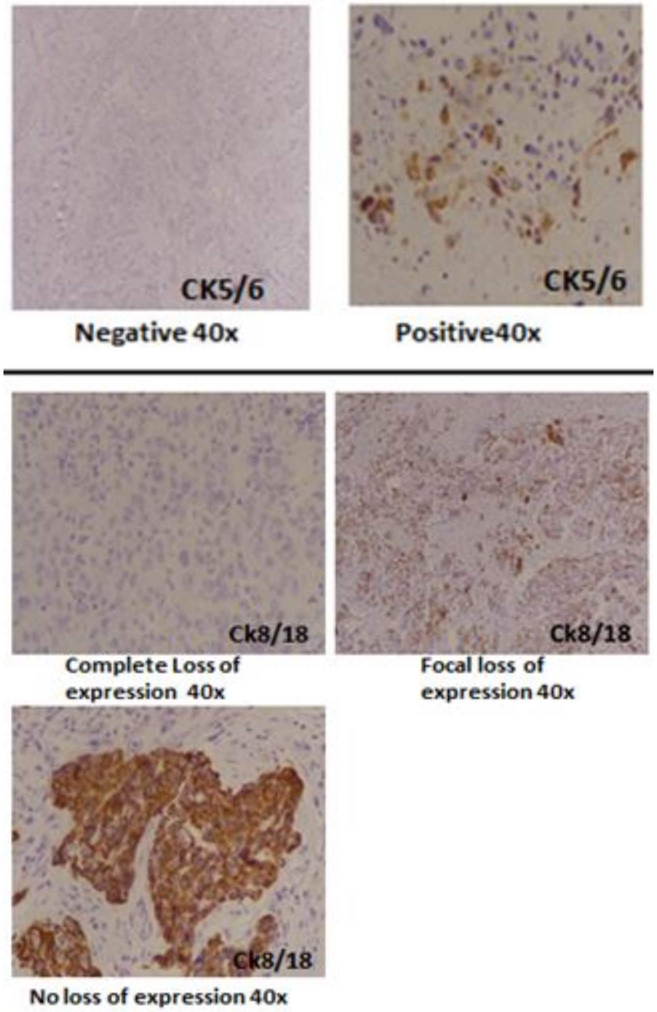
CK5/6
Negative 40x
CK5/6
Positive40x
Ck8/18
Complete Loss of
expression 40x
Ck8/18
Focal loss of
expression 40x
Ck8/18
No loss of expression 40x
