# Supplementary figures and images for: Cytokeratin 5/6 and cytokeratin 8/18 expression in triple negative breast cancers: clinicopathologic significance in South-Asian population
Source: BMC Res Notes. 2018 Jun 8;11:372. doi: 10.1186/s13104-018-3477-4 (PMC5994131; doi:10.1186/s13104-018-3477-4)

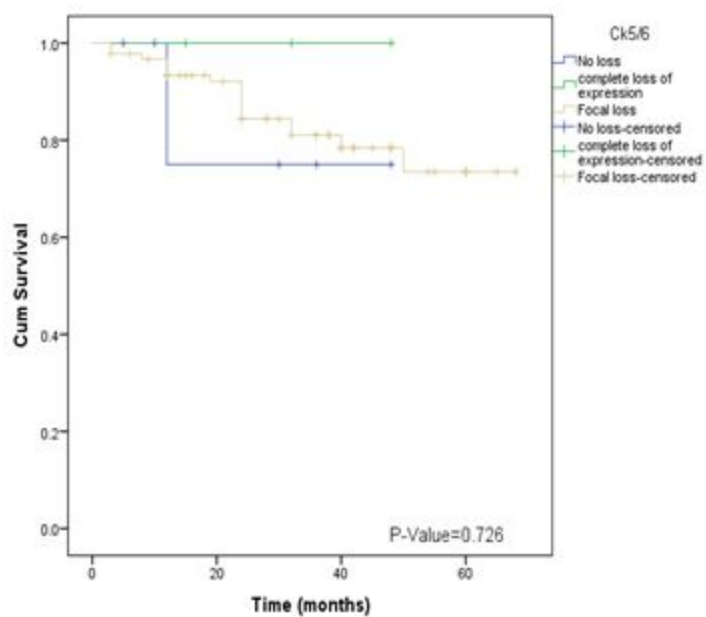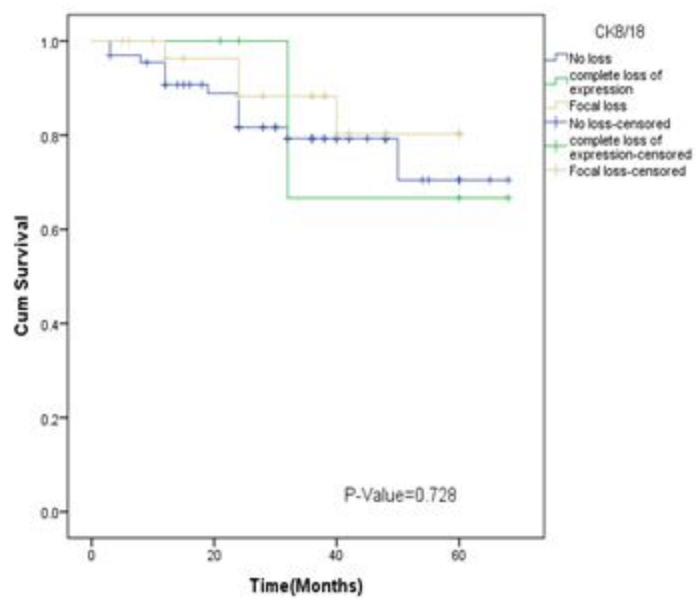

Supplement: Supplementary file 2 — Additional file 2: Figure S2. Kalpien-Meier curve (disease free survival) for CK 8/18 expression in triple negative breast cancer. [file 13104_2018_3477_MOESM2_ESM.pdf]
